# One-view digital breast tomosynthesis as a stand-alone modality for breast cancer detection: do we need more?

**DOI:** 10.1007/s00330-017-5167-3

**Published:** 2017-12-11

**Authors:** Alejandro Rodriguez-Ruiz, Albert Gubern-Merida, Mechli Imhof-Tas, Susanne Lardenoije, Alexander J. T. Wanders, Ingvar Andersson, Sophia Zackrisson, Kristina Lång, Magnus Dustler, Nico Karssemeijer, Ritse M. Mann, Ioannis Sechopoulos

**Affiliations:** 1 Department of Radiology and Nuclear Medicine, Radboud University Medical Centre, Geert Grooteplein 10, Post 766, 6525 GA Nijmegen, The Netherlands; 2 Bevolkingsonderzoek Zuid-West Borstkanker, Laan 20, 2512 GB Den Haag, The Netherlands; 3 Unilabs Breast Center, Skåne University Hospital, Jan Waldenströms gata 22, SE-20502 Malmö, Sweden; 4 Diagnostic Radiology, Department of Translational Medicine, Lund University, Skåne University Hospital, SE-20502 Malmö, Sweden; 5 Dutch Reference Centre for Screening (LRCB), Wijchenseweg 101, 6538 SW Nijmegen, The Netherlands

**Keywords:** Digital breast tomosynthesis, Digital mammography, Breast cancer, Receiver operating characteristic, Jack-knife alternative free-response receiver operating characteristic

## Abstract

**Purpose:**

To compare the performance of one-view digital breast tomosynthesis (1v-DBT) to that of three other protocols combining DBT and mammography (DM) for breast cancer detection.

**Materials and methods:**

Six radiologists, three experienced with 1v-DBT in screening, retrospectively reviewed 181 cases (76 malignant, 50 benign, 55 normal) in two sessions. First, they scored sequentially: 1v-DBT (medio-lateral oblique, MLO), 1v-DBT (MLO) + 1v-DM (cranio-caudal, CC) and two-view DM + DBT (2v-DM+2v-DBT). The second session involved only 2v-DM. Lesions were scored using BI-RADS® and level of suspiciousness (1–10). Sensitivity, specificity, receiver operating characteristic (ROC) and jack-knife alternative free-response ROC (JAFROC) were computed.

**Results:**

On average, 1v-DBT was non-inferior to any of the other protocols in terms of JAFROC figure-of-merit, area under ROC curve, sensitivity or specificity (p>0.391). While readers inexperienced with 1v-DBT screening improved their sensitivity when adding more images (69–79 %, p=0.019), experienced readers showed similar sensitivity (76 %) and specificity (70 %) between 1v-DBT and 2v-DM+2v-DBT (p=0.482). Subanalysis by lesion type and breast density showed no difference among modalities.

**Conclusion:**

Detection performance with 1v-DBT is not statistically inferior to 2v-DM or to 2v-DM+2v-DBT; its use as a stand-alone modality might be sufficient for readers experienced with this protocol.

****Key points**:**

*• One-view breast tomosynthesis is not inferior to two-view digital mammography.*

*• One-view DBT is not inferior to 2-view DM plus 2-view DBT.*

*• Training may lead to 1v-DBT being sufficient for screening.*

## Introduction

Digital breast tomosynthesis (DBT) improves breast cancer detection and diagnosis compared to digital mammography (DM) [1–9]. Instead of resulting in a two-dimensional (2D) image of the compressed breast as in DM, DBT acquires several low-dose 2D projections over a limited angular range, which are then used to reconstruct a pseudo-3D image of the breast [10]. Therefore, DBT can ameliorate the main limitation of DM: the anatomical noise due to tissue superposition. After years of promising results pointing to its superiority to DM, DBT is now being considered to be used for population-based breast cancer screening [11]. However, there is no agreement regarding how to implement DBT in screening.

Some implementations of DBT involve its use as an adjunct to DM. The main arguments for performing DM in addition to DBT are comparisons with prior mammograms, as well as results of some early studies that suggest that DBT is inferior for calcification detection and characterization [12, 13]. However, the primary negative effects of performing both modalities are an increase in reading time [14, 15] and in radiation dose [16]. The replacement of DM with synthetic 2D mammograms generated from DBT volumes allows for comparison with priors and reduces the radiation dose, while providing similar diagnostic performance to DM [17–19]. With regard to calcification detection, new studies indicate that DBT is not inferior to DM even for *wide-scan* angle DBT systems (those acquiring the projection images over an angular range of 40–50°, more prone to blurring due to a more oblique x-ray incidence) [20]. Thus, the ongoing evolution of the technique suggests the possibility of using DBT as a stand-alone modality.

However, the vast technical differences among commercial systems may need to be considered [10]. Using a *wide-scan* angle DBT system yields improved depth information [21]. Therefore, these systems better exploit the 3D advantage of DBT over DM, and may make the acquisition of two views of the breast unnecessary. A large screening trial has recently shown that using only the medio-lateral oblique (MLO) view of a *wide-scan* angle DBT system as a one-view stand-alone technique resulted in a 43 % breast cancer detection increase compared to two-view DM [7], supporting earlier pilot studies [22, 23]. Other preliminary studies also showed that one-view DBT is not significantly different to two-view DM, but they used a subject sample that was enriched, either totally [24, 25] or partially [26], with lesions detected with standard 2v-DM, therefore only allowing for a determination of non-inferiority for DBT.

In our study, we aim to further explore the potential of one-view DBT for breast cancer detection in screening, by performing a retrospective reader study with an enriched case dataset, comparing the clinical performance of one-view DBT (1v-DBT, MLO) with three other protocols: 1v-DBT (MLO) plus 1v-DM (CC), two-view DM (2v-DM) and 2v-DM plus 2v-DBT, something not yet reported in the same study. Furthermore, we also evaluate the strengths of 1v-DBT as a standalone technique stratified by lesion type, breast density and radiologist experience in reading 1v-DBT in a screening setup, which has not yet been studied.

## Methods

### Study population

This retrospective study was approved by the regional ethics board after summary review, with waiver of a full review and informed consent. The study used DM and DBT images from 181 women (median age 52 years, range 30–88 y) imaged at our hospital between December 2014 and December 2015, who were recalled from screening (33 %) or had a clinical indication for imaging (67 %). All the available cancer cases within the collection date were included, while benign and normal cases were consecutively included to meet the proportions described below. Women with a prior history of breast cancer or for whom the required views for this study were unavailable were excluded (in total, 25). Within the cohort, 76 patients had malignant lesions and 50 had benign lesions, all verified by histopathology. Four patients had multiple lesions, three patients with two different malignant lesions and one patient with one benign and one malignant lesion. In total, 130 lesions were diagnosed with histopathological proof (79 malignant, 51 benign). The remaining 55 patient cases were interpreted as normal (Breast Imaging Reporting and Data System, BI-RADS®, score 1 or 2), and had at least 1 year of negative imaging follow-up (mean follow-up: 378 days). Detailed characteristics of patient cases and identified lesions are presented in Table 1. Density according to BI-RADS® 5th Edition was obtained from the radiological case report from clinical routine.

**Table 1:** Characteristics of the patients and the lesions included in the observer study

Patient characteristics			
Cases (recalls from screening)		181 (60, 33 %)	
Normal		55 (0, 0 %)	
Biopsied benign		50 (24, 48 %)	
Biopsied malignant		76 (36, 47 %)	
Age			
Median age (y)		52 (range 30–88)	
< 40		15 (8 %)	
40–49		51 (28 %)	
50–59		66 (37 %)	
60–69		33 (18 %)	
≥ 70		16 (9 %)	
BI-RADS Breast density			
Almost entirely fatty		21 (12 %)	
Scattered fibroglandular densities		78 (43 %)	
Heterogeneously dense		62 (34 %)	
Extremely dense		20 (11 %)	
Biopsied lesion characteristics			
Total biopsied lesions		130	
Benign and high-risk lesions		51	
Histology		Lesion type	
Fibroadenoma	17	With soft tissue	30
Hyperplasia	10		
Fibrocystic changes	6		
Single papilloma	3	With calcifications	24
Lobular carcinoma in situ	3		
Others*	12		
Malignant lesions		79	
Histology		Lesion type	
Invasive ductal carcinoma	40	With soft tissue	58
Ductal carcinoma in situ	18		
Invasive lobular carcinoma	13	With calcifications	36
Invasive papillary carcinoma	3		
Others†	5		

*Examples are inflammation, adenosis, duct ectasia and benign phylloides tumour

†Examples are tubular carcinoma, non-Hodgkin’s lymphoma, and neuroendocrine carcinoma

### Patient images

All patients had undergone diagnostic breast imaging using a commercial DBT system (Mammomat Inspiration, Siemens, Erlangen, Germany). For each patient 2v-DM and 2v-DBT (CC and MLO) were obtained. For DBT, the system acquires 25 low-dose projection images over an angular range of approximately 50° (*wide-angle* breast tomosynthesis) in about 25 s [10]. The projection images are subsequently reconstructed using a filtered back-projection algorithm [27]. Four different unilateral (breast with the most suspicious findings, or randomly selected if normal) image sets resembling four different imaging protocols were created for each patient: one-view breast tomosynthesis (1v-DBT, MLO), 1v-DBT (MLO) plus 1v-DM (CC), 2v-DM and 2v-DM plus 2v-DBT.

### Study design

**Fig. 1:**
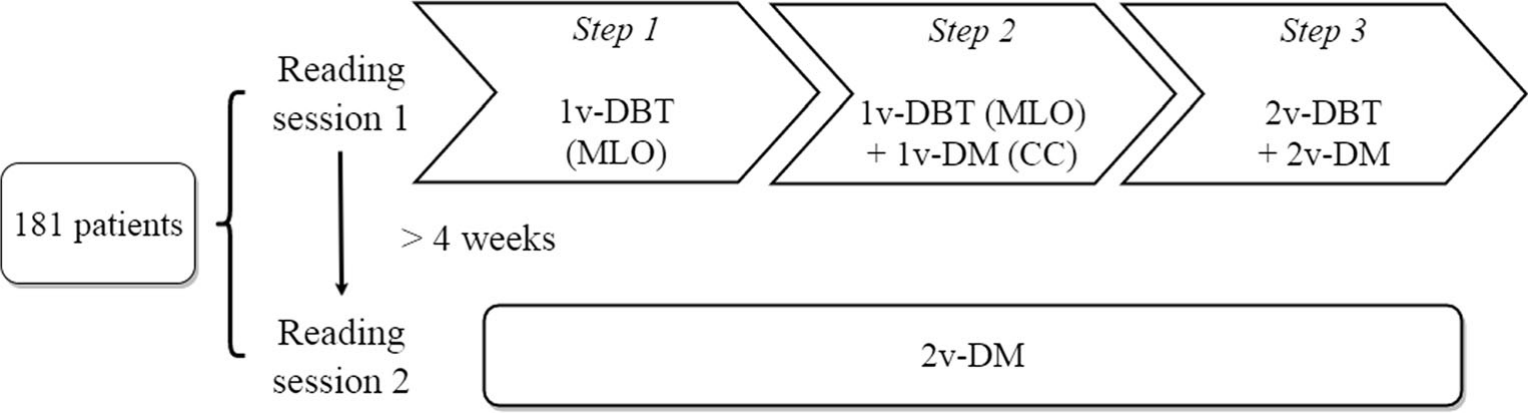
Schematic of the study design, where 1v-DBT (MLO) was compared to 1v-DBT (MLO) + 1v-DM (CC), to 2v-DBT + 2v-DM, and with respect to 2v-DM. The study was carried out in two different reading sessions, the first one sequential, the second one at least 4 weeks later

A retrospective reader study consisting of two reading sessions (Fig. 1) was designed to evaluate the detection performance of the four protocols. The first session had a sequential design with three distinct steps per patient, where the readers were shown progressively 1v-DBT (MLO) (step 1), then 1v-DBT (MLO) plus 1v-DM (CC) (step 2) and finally 2v-DM plus 2v-DBT (step 3). The second reading session was performed at least 4 weeks (considered enough for a memory washout period) after the first session and consisted of only the 2v-DM images. A training set consisting of 20 cases was reviewed to begin each session. The readers were blinded to any information about the patient and any prior imaging.

At each step, the reader was asked to annotate and score all the detected suspicious lesions. For each abnormality, the observer gave two scores: a forced BI-RADS® assessment (1 – normal, 2 – benign findings, 3 – low probability of malignancy, 4 - suspicious of malignancy, 5 – highly suspicious of malignancy) as established by the American College of Radiology, and a level of suspiciousness score (10-point scale, from 1 – high probability of benign; to 10 – high probability of malignancy).

The experiment was performed on an in-house developed workstation (CIRRUS Observer, Diagnostic Image Analysis Group, Nijmegen, The Netherlands) using dual 5-MP or one 10-MP mammographic display(s). The workstation also automatically recorded reading times for each modality.

### Reference standard

The reference standard, including location and lesion type, was established by one radiologist who did not participate in the study and had 13 years of experience in DM and 2 in DBT, with knowledge of the clinical presentation, additional imaging tests including priors and histopathology reports, when available.

### Readers

Six breast radiologists performed the study. They are part of three different institutions across two countries: The Netherlands and Sweden. We recognized two distinct categorical groups of readers; the three readers from The Netherlands, who had no experience in reading 1v-DBT as a stand-alone modality for breast cancer screening, and the three readers from Sweden, who were experienced in this approach due to participation in a large 1v-DBT screening trial [7]. The three experienced readers with 1v-DBT had 3, 12 and 44 years of experience with mammography and 3, 9 and 10 years of experience with DBT. The three inexperienced readers with 1v-DBT had 17, 26 and 35 years of experience with mammography and 2, 2 and 3 years of clinical experience with DBT in combo mode (2v-DM + 2v-DBT).

### Statistical analysis

Four different analyses were performed. First, for a precise evaluation, a jack-knife alternative free-response receiver operating characteristic (JAFROC) analysis was performed [28]. For this, the lesion localizations by the readers (considered correct if within 2 cm of the reference standard) and the level of suspiciousness were used. JAFROC provides a figure of merit (FoM) defined as the probability that a correctly marked lesion is rated higher than the highest-rated mark on a normal/benign case [29].

For the second analysis, receiver operating characteristic (ROC) curves and their area under the curve (AUC) were computed. Since ROC analysis requires that diagnostic confidence is expressed in an ordinal scale, level of suspiciousness and not BI-RADS® scores of the most suspicious finding per case were used [30]. ROC analysis was repeated discriminating by lesion type (soft tissue lesions or calcifications; if a lesion was composed of both types, it was counted on each category) and breast density category (low, *a* and *b*; or high, *c* and *d*).

Significance testing of ROC and JAFROC was performed using the Dorfman–Berbaum–Metz multiple reader, multiple-case mixed-model analysis of variance, which yields a p-value for rejecting the null hypothesis that the four modalities have equal performance. Random-reader and random-case analysis was performed [28, 29, 31].

To study the impact that our results would have in a screening scenario, sensitivity and specificity on a per-case basis were computed using BI-RADS® categories, using BI-RADS® category 3 or higher defined as a positive interpretation. Cases with biopsied benign lesions were considered as false positives if they were rated positive by the readers. Average sensitivity and specificity for all imaging protocols were computed using a generalized linear model (GLM) to account for multiple reader, multiple case repeated measures. Parameter estimates of the GLM were bootstrapped (n=1,000). The all two-way GLM model was built with an unstructured covariance matrix, using modality and reader as factors. To adjust for multiple comparison, the least significant difference correction was used. Model-based Wald 95 % confidence intervals were calculated. Statistical significance among modalities for each reader was estimated using McNemar’s paired test.

Finally, reading times, defined as the time spent on evaluating, scoring and annotating 1v-DBT (first step of the first reading session) and 2v-DM (second reading session), were compared using two-way repeated measures ANOVA. Outliers, defined as times whose values extended beyond 1.5 standard deviations, were removed. Also, mean glandular doses were retrieved from the DICOM (Digital Imaging and Communications in Medicine) headers for comparison. Differences between modalities for each reader were compared using a paired Student’s t-test.

A two-tailed *p* value lower than 0.05 was considered to indicate significant difference. All analyses were performed using SPSS (version 24, IBM Inc., Armonk, NY, USA) and open-access JAFROC software by Dev Chakraborty (version 4.2.1, DevChakraborty.com).

## Results

**Fig. 2:**
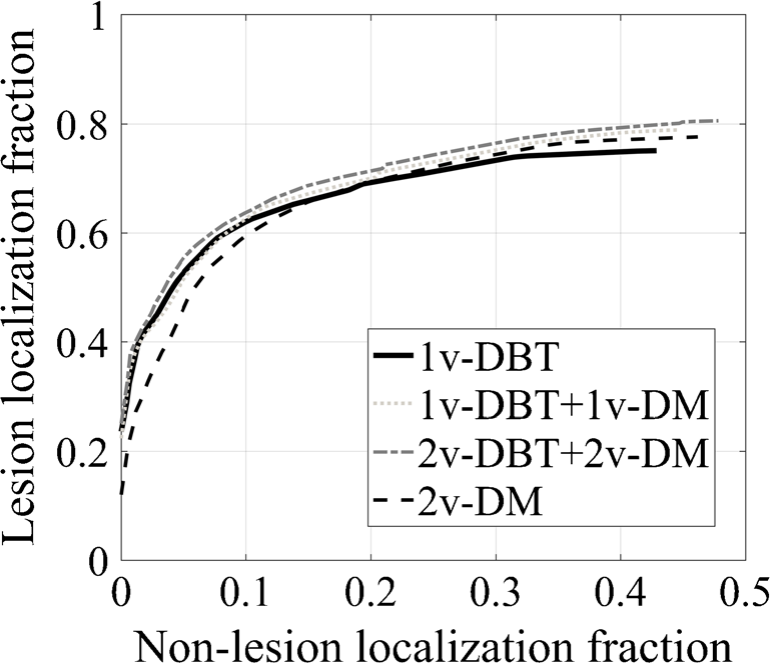
JAFROC analysis averaged for all readers for each reading protocol. The lesion localization fraction is the number of correctly identified lesions divided by total number of lesions (0 ≤ LLF ≤ 1), while the non-lesion localization fraction is the number of marks which are not close to any lesions, divided by total number of images (0 ≤ NLF); note the lack of an upper bound

The JAFROC curves averaged for all readers are shown in Fig. 2, while individual JAFROC FoM per reader and experience are shown in Table 2. There was no statistical difference between 1v-DBT and the other protocols (p=0.522), either on average or per reader. The FoM for 1v-DBT was similar to 2v-DM and slightly lower than for the rest, while it was comparable between experienced and inexperienced readers with 1v-DBT. Only a small difference was found in the JAFROC FoM between experienced and inexperienced observers, the latter performing slightly better with 2v-DM. Relative results did not change if the biopsied benign cases were removed from the analysis, and 1v-DBT yielded similar performance to the other three modalities (p=0.459).

**Table 2:** JAFROC (jack-knife alternative free-response receiver operating characteristic) figure of merit (FoM) per reader and by reader group according to experience. The FoM is defined as the probability that a malignant lesion is rated higher than any mark on an image which does not contain malignancies (95 % CI is shown within parentheses)

Reader	1v-DBT	1v-DBT+1v DM	2v-DBT+2v-DM	2v-DM
R1	0.782	0.788	0.805	0.759
R2	0.764	0.757	0.785	0.799
R3	0.731	0.760	0.760	0.770
R4 (exp)	0.767	0.773	0.784	0.773
R5 (exp)	0.745	0.763	0.760	0.717
R6 (exp)	0.774	0.788	0.796	0.760
Inexperienced with 1v-DBT	0.759 (0.692–0.825)	0.768 (0.706–0.831)	0.783 (0.720–0.847)	0.776 (0.709 –0.843)
Experienced with 1v-DBT	0.762 (0.700–0.825)	0.775 (0.713–0.837)	0.780 (0.717–0.844)	0.750 (0.680–0.820)
All	0.761 (0.699–0.822)	0.772 (0.711–0.832)	0.782 (0.721–0.842)	0.763 (0.699–0.827)

**Fig. 3:**
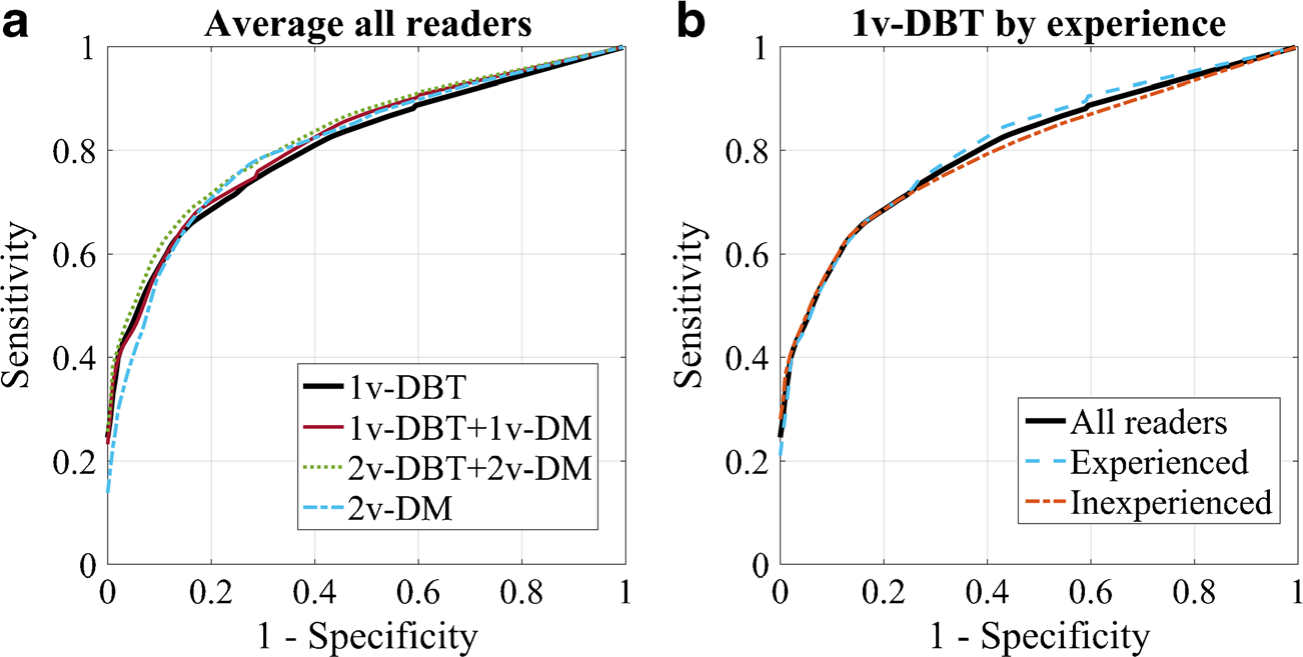
Average receiver operating characteristic (ROC) curves computed with the level of suspiciousness score of the highest rated lesion on each case: (**a**) for each imaging protocol considering all readers and (**b**) for 1v-DBT differentiating by groups of experience with this protocol among readers

The average radiologist’s ROC curve is shown in Fig. 3a for each imaging protocol. The AUC of 1v-DBT was not statistically significantly different than that of the other protocols for the average of readers (p=0.391, Table 3 and Fig. 3a). Only in two cases was 1v-DBT significantly different with respect to another protocol: for one reader (experienced with 1v-DBT), the AUC was statistically better for 1v-DBT than for 2v-DM (p=0.011), while for another reader (inexperienced with 1v-DBT) 1v-DBT performed worse than 2v-DM (p=0.035). Experienced readers had only slightly higher AUCs for 1v-DBT than the inexperienced readers (Fig. 3b; experienced = 0.815 (CI: 0.760-0.871), inexperienced = 0.800 (CI: 0.746-0.855), not significant, p=0.775). On the other hand, AUC for 2v-DM was lower (p=0.425) in the experienced (0.793, CI: 716-0.871) compared to the inexperienced group (0.831, CI: 775-0.887).

**Table 3:** Area under the curve of the average receiver operating characteristic (ROC) curve for each of the studied imaging protocols. Parentheses indicate 95 % confidence intervals

	1v-DBT	1v-DBT+1v DM	2v-DBT+2v-DM	2v-DM
Average reader	0.808 (0.754–0.862)	0.818 (0.766–0.870)	0.830 (0.778–0.883)	0.812 (0.752–0.872)

Using ROC analysis, no significant difference was found between 1v-DBT and the other modalities either by separating the cases by breast density (low, p=0.601; or high, p=0.323), or by separating the lesions by type (soft tissue, p=0.329; or calcifications, p=0.499). It was also seen that 1v-DBT performs better than 2v-DM at low false-positive rates, both in ROC (Fig. 3a) and JAFROC (Fig. 2) analyses.

**Fig. 4:**
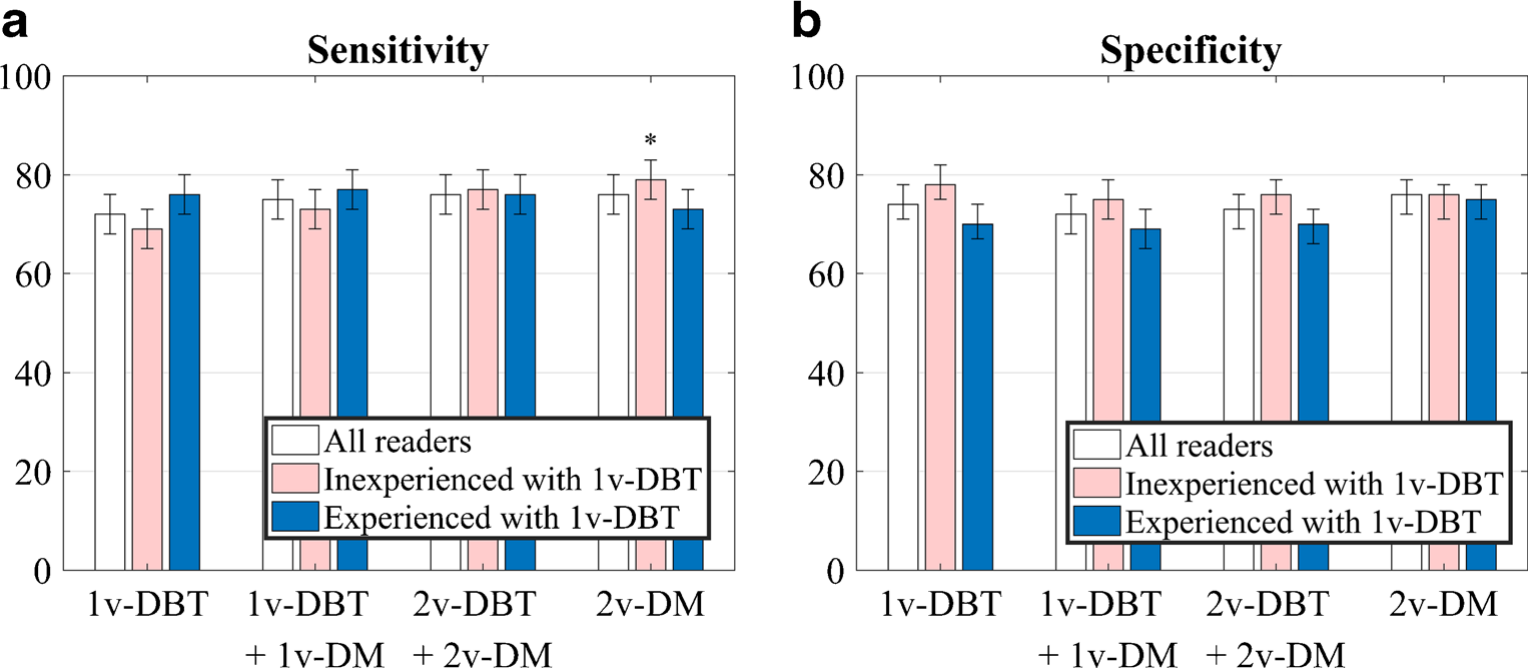
Average (**a**) sensitivity and (**b**) specificity (in %) on each imaging protocol, for all the readers as well as differentiating by level of experience. Error bars indicate Wald 95 % confidence intervals. Significant differences with respect to 1v-DBT are indicated with a (*)

The sensitivity and specificity per reader and on average for each imaging protocol is shown in Table 4 and Fig. 4. No difference was found between 1v-DBT and the other modalities either for sensitivity (p=0.536) or specificity (p=0.553). There were differences in the results among the six readers (p<0.001). For the group of 1v-DBT experienced radiologists, there was no statistically significant difference either in sensitivity (p=0.776) or specificity (p=0.482) between 1v-DBT and the other protocols. For the inexperienced group, sensitivity increased for all the other protocols with respect to 1v-DBT (only significant for 2v-DM, from 69 % to 79 %, p=0.019), while specificity was slightly higher for 1v-DBT with respect to the other protocols (not significant, p=0.777).

**Table 4:** Sensitivity and specificity (in %, within parentheses 95 % Wald confidence intervals) for the average of all readers and grouped by experience, using the BI-RADS® score of the most suspicions finding on each case

Reader	1v-DBT	1v-DBT+1v DM	2v-DBT+2v-DM	2v-DM
Sensitivity				
R1	78 (68–87)	83 (74–92)	82 (73–90)	83 (74–92)
R2	66 (55–77)	71 (61–81)	76 (67–86) *	79 (70–88) *
R3	63 (52–74)	65 (53–75)	72 (62–83) *	74 (64–84) *
R4 (exp)	78 (68–87)	78 (68–87)	80 (71–89)	75 (65–85)
R5 (exp)	68 (58–79)	72 (62–83)	67 (56–78)	65 (53–75)
R6 (exp)	80 (71–89)	79 (70–88)	79 (70–88)	78 (68–87)
All	72 (68–76)	75 (71–79)	76 (72–80)	76 (72–80)
Specificity				
R1	66 (56–75)	63 (53–72)	67 (58–76)	63 (53–72)
R2	84 (77–91)	79 (71–87)	84 (77–81)	82 (74–89)
R3	83 (76–90)	80 (72–88)	75 (67–84) *	82 (74–89)
R4 (exp)	68 (59–77)	66 (56–75)	65 (55–74)	73 (65–83)
R5 (exp)	77 (69–85)	76 (68–84)	78 (70–86)	78 (70–86)
R6 (exp)	66 (56–75)	66 (56–75)	67 (58–76)	71 (63–80)
All	74 (71–78)	72 (68–76)	73 (69–76)	76 (72–79)

*Significant (p-value <0.05) difference with respect to 1v-DBT

**Fig. 5:**
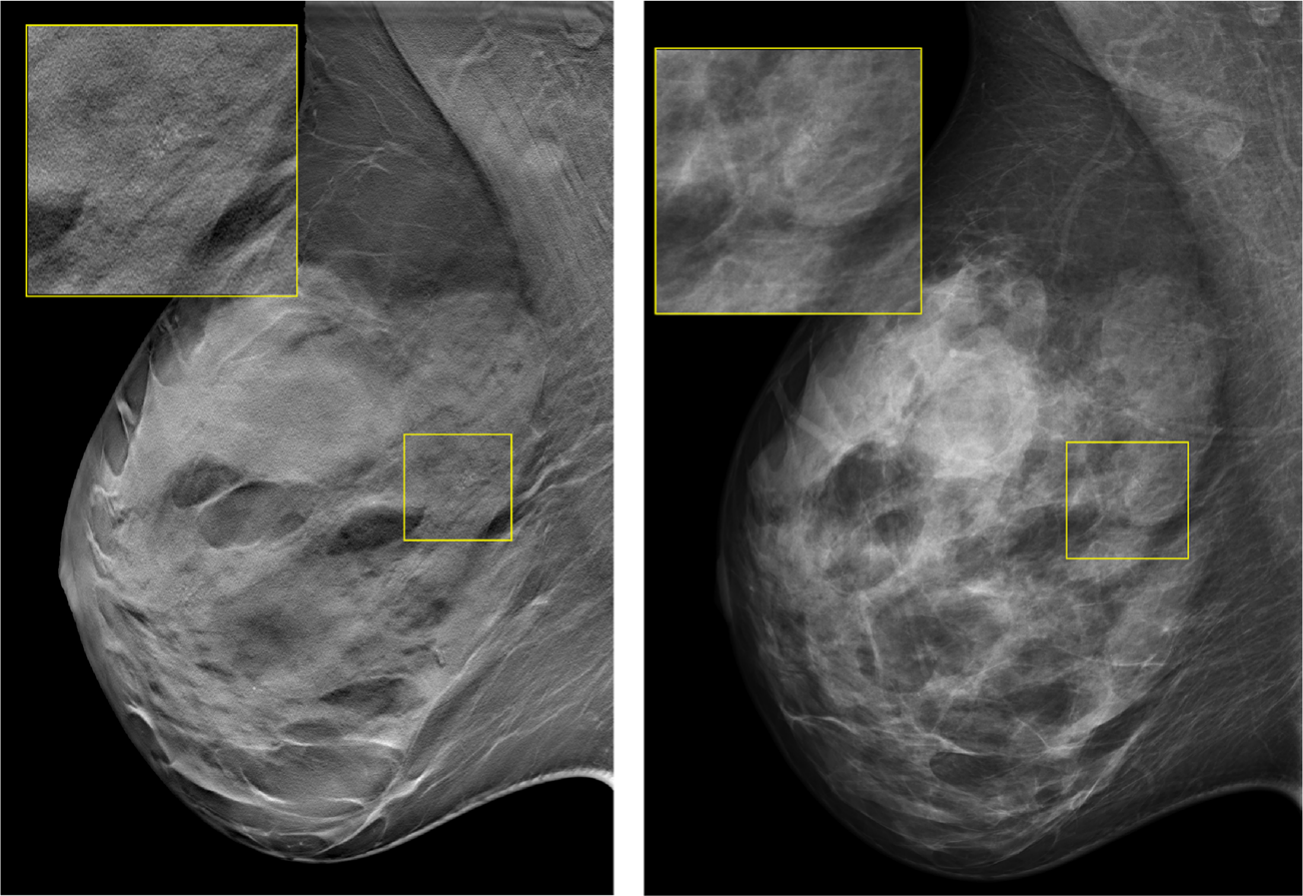
Example of a patient with a ductal carcinoma in situ grade II. This case was recalled by three readers and two readers with 1v-DBT and 2v-DBT/2v-DM, respectively, and it was not recalled by any reader with 2v-DM: (i) MLO tomosynthesis slice where the lesion is in focus. (ii) MLO mammography

**Fig. 6:**
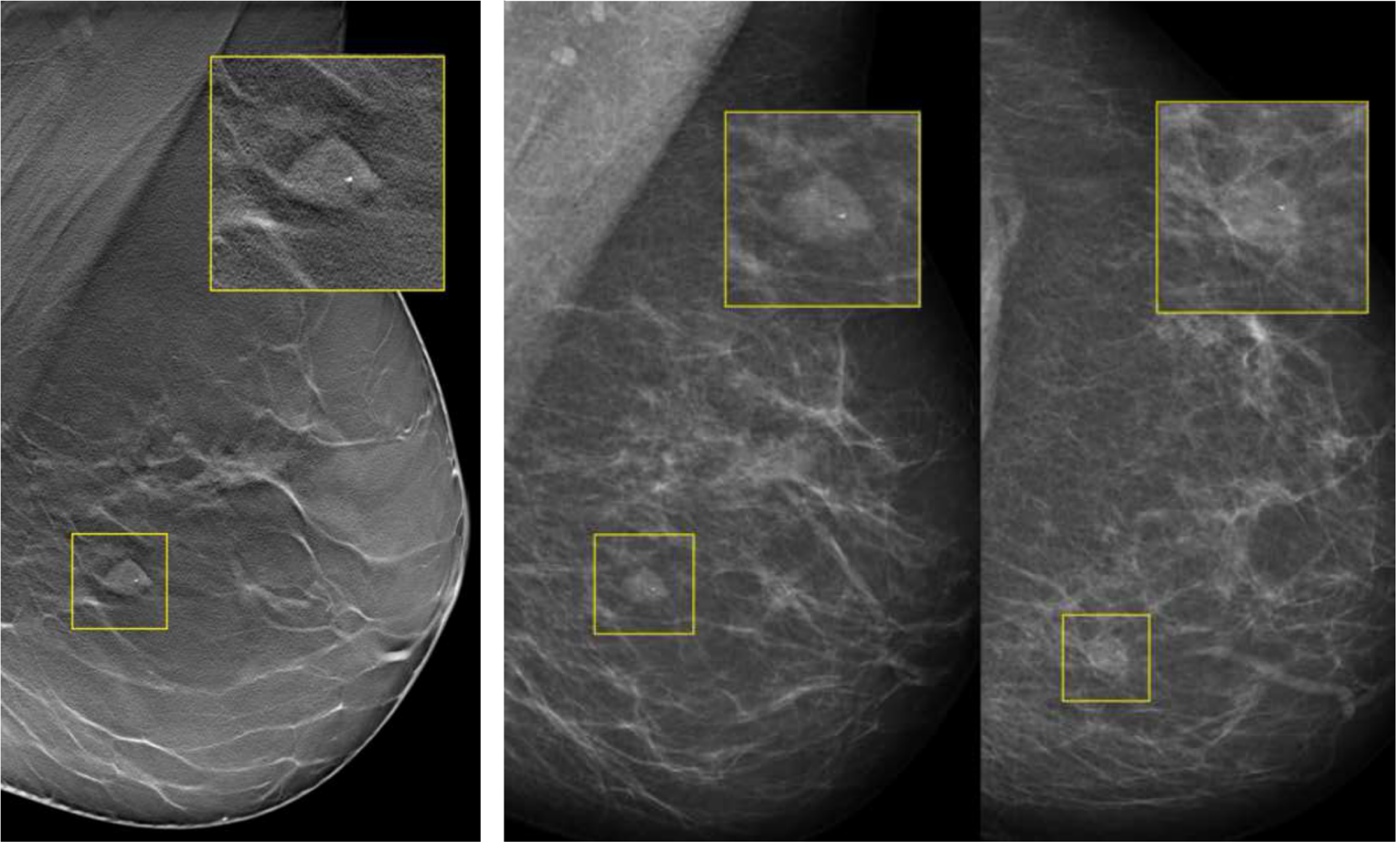
Example of a patient with a sclerosed fibroadenoma, recalled by one reader with 1v-DBT, by one reader with 2v-DBT/2v-DM, and recalled by all six readers with 2v-DM: (i) MLO tomosynthesis slice where the lesion is in focus. (ii) MLO and CC mammograph

**Fig. 7:**
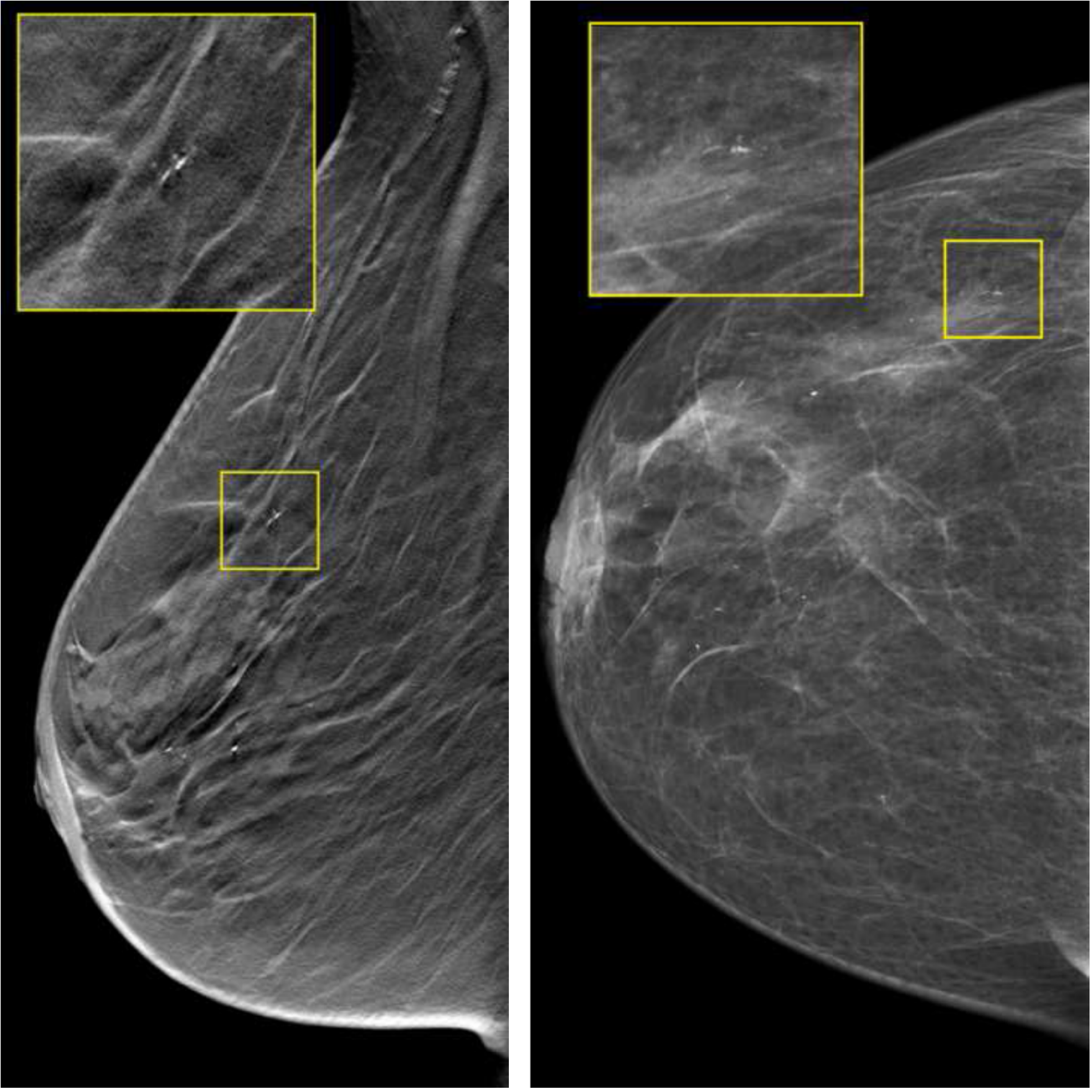
Example of a patient with an invasive ductal carcinoma with ductal carcinoma in situ grade II, who was recalled by only one reader with 1v-DBT, by four readers with 2v-DBT/2v-DM, and by all six readers with 2v-DM: (i) MLO tomosynthesis slice where the lesion is in focus. (ii) CC mammography

**Fig. 8:**
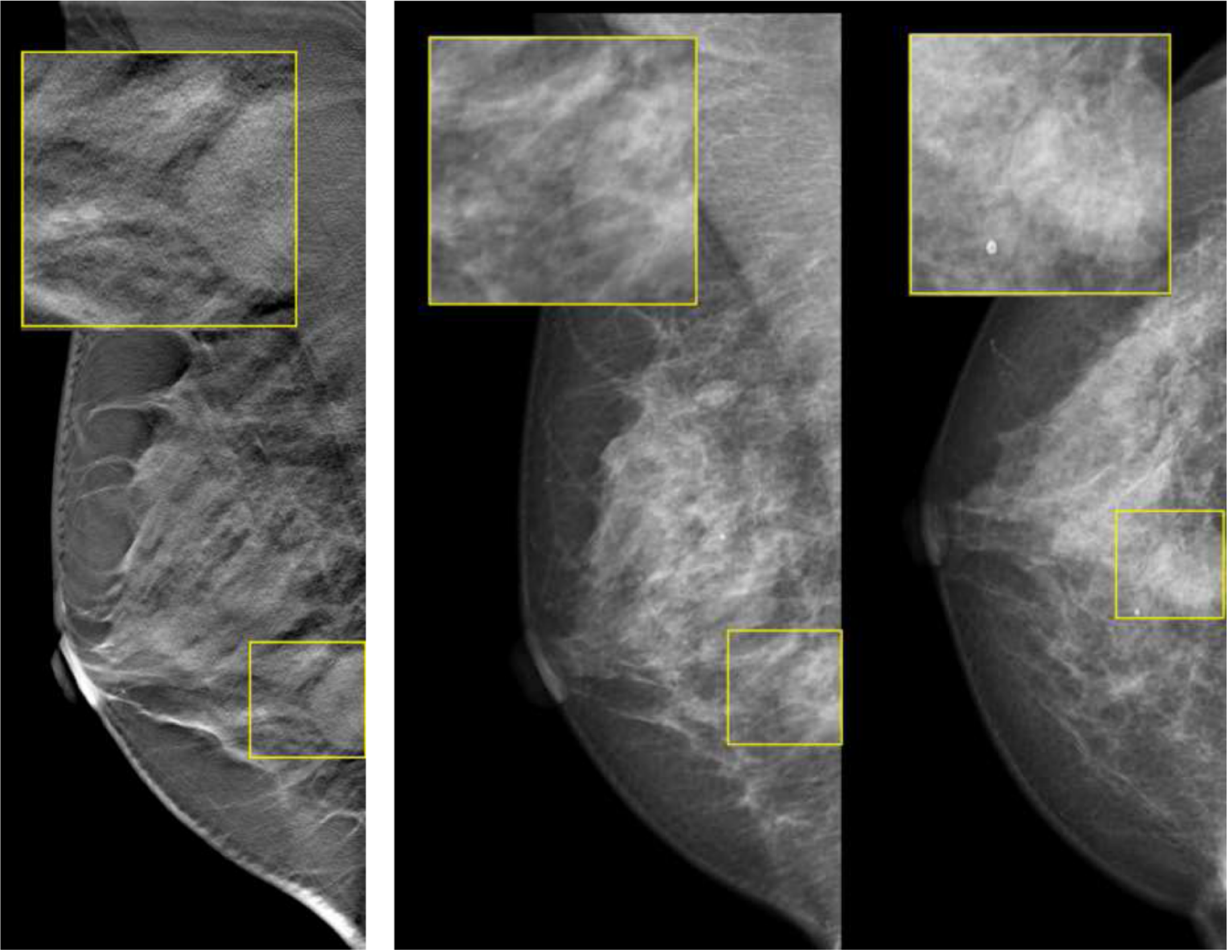
Example of a patient with a fibroadenoma, recalled by four readers with 1v-DBT, by three readers with 2v-DBT/2v-DM, and only recalled by one reader with 2v-DM (all readers marked the lesion in all the modalities): (i) MLO tomosynthesis slice where the lesion is in focus. (ii) MLO and CC mammography

Two examples of cases that were correctly assessed by most readers in 1v-DBT and not in 2v-DM are shown in Figs. 5 and 6, while two cases assessed correctly by most readers in 2v-DM and not in 1v-DBT are displayed in Figs. 7 and 8. Apparently, the effect DBT can have in benign lesions is bidirectional (Figs. 6, and 8), sometimes leading to increased suspiciousness and recall and sometimes reducing suspiciousness and avoiding recall.

The mean glandular dose per study was equal between 1v-DBT (2.41 ± 0.87 mGy) and 2v-DM (2.41 ± 0.83 mGy). The mean dose for 1v-DBT+1v-DM was 3.62 ± 1.25 mGy and for 2v-DBT+2v-DM was 7.23 ± 2.49 mGy. The average reading time was higher for 1v-DBT with respect to 2v-DM (55 s versus 44 s, p<0.001, see Table 5). For three readers there was no statistical difference between 1v-DBT and 2v-DM, and no difference in reading time was found between experienced and inexperienced observers. A total of 41 reading time outliers were identified among all readers (median six outliers per reader, range 2–15).

**Table 5:** Reading time (in seconds, mean value and 95 % CI within parentheses) for each reader and on average, compared between 1v-DBT and 2v-DM. Outliers greater than 1.5 times the standard deviation of the data were removed

Reader	1v-DBT	2v-DM	p-value
R1	58 (52–65)	36 (32–40)	<0.001
R2	57 (52–63)	51 (46–57)	0.095
R3	42 (37–47)	46 (40–53)	0.281
R4 (exp)	63 (55–71)	64 (56–71)	0.880
R5 (exp)	56 (50–62)	31 (27–35)	<0.001
R6 (exp)	55 (48–62)	35 (30–40)	<0.001
Average	55 (52–59)	44 (40–48)	<0.001

## Discussion

The results of our study suggest that one-view digital breast tomosynthesis is not significantly different than two-view digital mammography and the combination of two-view mammography plus two-view tomosynthesis for breast cancer detection.

The addition of 1v-DM (CC) to 1v-DBT (MLO), one of the protocols recommended by some manufacturers, yielded an increase in sensitivity but also a small decrease in specificity for the inexperienced readers. For the readers experienced with 1v-DBT, no increase in sensitivity was found when adding 1v-DM to 1v-DBT, similar to the results by Lång et al. [7]. In general, for experienced readers, 1v-DBT proved to be enough in terms of sensitivity and specificity, and no added value was found with extra views. The results of the inexperienced 1v-DBT reader group were different. These radiologists operate at a different point along the same ROC curve as the experienced 1v-DBT reader group, either due to local screening practices or due to their not being accustomed to arriving at a decision with a single view. Overall, they had a higher specificity and lower sensitivity for 1v-DBT, that respectively decreased and increased when more images were added. The higher specificity could be explained due to having more experience in reading mammograms. However, their performance in terms of ROC was similar to that of the experienced readers, suggesting that training could lead them to operate at the same point as the more experienced readers with 1v-DBT.

These results are similar when taking lesion localization into account and computing the figure-of-merit of the JAFROC analysis. Nevertheless, as could be expected, the protocol with more images available for the radiologist, 2v-DBT plus 2v-DM, yielded a slightly better, but not significant, performance. We also saw that 1v-DBT performs better than 2v-DM at low false positives, which could be particularly relevant and important for screening.

The experience level with mammography might have also played a role in our results. As suggested by some studies, the least experienced readers with mammography benefit the most from using DBT [26, 32]. In our case, we observed that the less experienced readers with mammography had a lower ROC performance with 2v-DM than the others, but similar performance with 1v-DBT.

When looking at different lesion types, we found that 1v-DBT is not statistically inferior to any other tested protocol for the task of detecting lesions with calcifications, which adds to the results by other authors [33, 26, 20] and suggests that DBT is not inferior for the detection of calcifications even with a *wide-angle* DBT system.

All these results suggest that the use of 1v-DBT as a stand-alone modality for breast cancer screening may be feasible, since the added value of the other DBT view or any DM views was not found significant in this study. Aside from the *Malmö Breast Tomosynthesis Screening Trial*, which was performed with 1v-DBT [7], most screening trials with DBT have used a protocol consisting of 2v-DBT with *narrow-angle* systems [3, 17, 18, 34, 19]. All studies report equivalent increases in breast cancer detection rates, and similar recall rates [11]. Screening is different from clinical practice. A mass screening policy always implies compromises due to constraints of costs, staffing, radiation dose to the population and other factors. In clinical practice, such constraints are less of an issue.

The Malmö Study showed a 43 % increase in cancer detection rate with 1v-DBT compared with 2v-DM. Clearly, adding a CC-view in DBT would increase the cancer detection rate marginally, just as e.g. adding breast ultrasound examination would do, something that is usually considered not feasible except in high risk groups. In the future, screening is in all likelihood going to be individualized based on risk profile. High-risk groups will probably be offered something increasing the sensitivity which may be another DBT view, ultrasound or even MRI, the latter already being the case in many programs for women with the highest risk. Nevertheless, in a screening scenario, we assume that if 2v-DBT is not feasible due to implementation reasons, there is an overall benefit in detection achieved by performing 1v-DBT instead of two-view DM. Finally, it is yet to be seen if 1v-DBT with a narrow-angle DBT system yields at least the same performance as 2v-DM for breast cancer detection, something not assessed in this work due to its single manufacturer limitation.

The increased reading time for DBT in comparison to DM is still one of the pitfalls that can be improved before implementing DBT in a screening setup [15, 35]. Certainly, using one-view instead of two-view DBT, without losing clinical performance, could ameliorate this problem. We observed that 1v-DBT took on average 25 % longer to read than 2v-DM (although for half of the observers reading times were equivalent). Yet, it is also possible that longer loading times of DBT in comparison with DM influenced reading times in our study. Additional training of radiologists on reading DBT, the inclusion of synthetic mammograms and computer aided detection systems might aid speeding the reading of DBT.

The main limitation of our study is the fact that around 50 % of the positive cases in our dataset are recalls from the Dutch DM-based screening program. Therefore, these were lesions already seen in 2v-DM. The lack of a true DBT screening population in our study, or at least enrichment with lesions first detected with either modality, leads to a bias towards 2v-DM, and thus the true benefit of 1v-DBT in screening might be larger than documented in our study. The real sensitivity and specificity of 1v-DBT in screening practice can only be assessed in a screening study, but our study in contrast allows determination of the relative differences in reading mode. We included all the cancer cases available at our institution, but the study could have benefitted from additional cases detected by DBT screening programs. Also, there were two different sets of radiologists involved in the readings, who might have different operating points based on local routine. Another minor limitation may be the stepwise nature of the first reading session, rather than dividing into three different sessions. However, we believe the stepwise scheme could introduce a bias, if at all, against 1v-DBT, which does not affect the conclusion of this work.

## Conclusion

Detection performance with 1v-DBT is not statistically inferior to the standard protocols of 2v-DM and 2v-DM+2v-DBT, and its use as a stand-alone modality might be sufficient for readers experienced with this protocol. Based upon the overall equivalent performance in terms of ROC and JAFROC analysis, experience with single-view DBT interpretation might change the operating point of radiologists, making their sensitivity/specificity performance in a screening scenario equivalent to that of two-view DM plus two-view DBT. Therefore, with a *wide-angle* system and appropriate training, MLO view-only DBT might be feasible for breast cancer screening.

## Abbreviations

1v-DBT: One-view digital breast tomosynthesis (MLO)
1v-DBT+1v-DM: One-view digital breast tomosynthesis (MLO) plus one-view digital mammography (CC)
2v-DM: Two-view digital mammography (CC + MLO)
2v-DBT+2v-DM: Two-view digital breast tomosynthesis (CC + MLO) plus two-view digital mammography (CC + MLO)
ANOVA: Analysis of variance
AUC: Area under the curve
BI-RADS: Breast imaging reporting and data system
CC: Cranio caudal
DBT: Digital breast tomosynthesis
DICOM: Digital imaging and communications in medicine
DM: Digital mammography
FOM: Figure of merit
JAFROC: Jack-knife alternative free-response receiver operating characteristic
MLO: Medio-lateral oblique
ROC: Receiver operating characteristic
